# Some of the Uses of Nitro-Glycerine*Read before the New York Neurological Society, October 4th, 1881.

**Published:** 1881-11-20

**Authors:** William A. Hammond

**Affiliations:** Surgeon-General U. S. Army (Retired List); Professor of Diseases of the Mind and Nervous System in the University of the City of New York, etc.


### SOME OF THE THERAPEUTICAL USES OF NITRO-GLYCERINE.*

BY WILLIAM A. HAMMOND, M. D.,

Surgeon-General U. S. Army (Retired List); Professor of Diseases of the Mind and Nervous System in the University of the City of New York, etc.

If a drop of solution of nitro-glycerine in alcohol, in the proportion of one part in a hundred, be placed on the tip of the tongue, a sensation of fullness and pain in the head (mainly in the frontal region) is experienced in the course of three or four minutes. This fullness disappears in a short time. A dose of three or four drops of the strength mentioned, produces headache of much greater severity and of longer duration. The carotid and temporal arteries pulsate with increased force; the head feels as if it is about to burst open; the face becomes red; the action of the heart is augmented and the respiration becomes more frequent. These symptoms are indicative of cardiac and vascular excitement, and of cerebral hyperæmia. We should therefore *a priori* expect that nitro glycerine would be useful in those cases in which it was desirable to stimulate the circulatory system and to increase the amount of the intra-cranial blood. For the last two years I have made frequent use of this very powerful agent, and it has occurred to me that the results of my experience might be of interest to the members of the Neurological Society and to the profession at large.

First, however, a few words in regard to the preparation to employ and the method of using it. There are great differences in nitro-glycerine (or glonoin, as it is sometimes called), as it is met with in the shops. Some specimeas of it are altogether inert, and some are of such extraordinary strength as to render their employment dangerous. I make use of that prepared by Bernicke & Tafel, in this city, which contains ten parts in every hundred, that is, ten of nitro-glycerine and ninety of absolute alcohol to the extent of ninety parts to ten of the drug. Thus:—

R. Nitro-glycerine (one-tenth)................minims xl.
Alcohol.................................... ℥vj,
M. Ft. sol.

One drop of this solution contains the one one-hundreth (100) of a drop of nitro glycerine, and I never begin the treatment of any case with a larger dose than a drop of this dilution.

Some apothecaries, as I have ascertained by actual experience, do not know anything about the agent. When it is prescribed, they send for it and get the pure substance which—regardless of the figures 1-10 on the prescription, the meaning of which they do not know—they proceed to add in the proportion of minims xl. to ℥vj of alcohol. The consequence is that a solution is formed, every drop of which contains the one-tenth of a drop of nitro-glycerine; such a dose is

*Read before the New York Neurological Society, October 4th, 1881.

calculated to produce very serious and painful symptoms not unattended with danger.

Again, they may produce a solution which already contains only one-hnndreth or even one-thousandth of the agent. If minims xl of such a dilution are still further diluted with ℥vj of alcohol, a degree of attenuation is reached which may be of some value to those with homœopathic proclivities, but which is altogether inert as a remedy.

It is necessary, therefore, for the physician employing nitro-glycerine to take special care that the strength of the solution is exactly what he wants; otherwise he may get very much more effect than is desirable, or none at all. It would perhaps be better for him to procure Bernicke & Tafel's one-tenth dilution and prepare his prescription himself.

MIGRAINE OR HERMICRANIA.—Du Bois Reymond is of the opinion that migraine is always the consequence of a spasmodic construction of the blood vessels of the brain, by which their calibre is diminished. Mollendorf, on the other hand, contended that there is a relaxation of the vessels. According to the one theory, there is in migraine cerebral anæmia; to the other, cerebral congestion. But it is quite certain that neither of these views is exclusively correct, and that both are partially so. Eulenberg and Gutman held that in some cases of migraine there is cerebral anæmia, due to a tetanoid condition of the muscular coat of the arteries, while in others there is cerebral hyperæmia or congestion, resulting from a paralysis of the muscular coat. Berger, in his excellent monograph, expresses the same opinion, and my own experience, which has been extensive, convinces me that there is no doubt that this latter theory is the correct one. Both clinical observation and the action of remedies show us that these are the two essentially distinct forms of this disease.

Without going into a detailed consideration of the diagnostic marks of the two varieties of migraine (for which I must refer the reader to the special works on the subject), I will only say that opthalmoscopic examination will generally indicate to us the nature of the particular attack with which we have to deal. In the congestive form, the fundus of the eye of the affected side is, as Mollendorf observes, of a bright scarlet color, while that of the sound side retains its ordinary brownish-red tint. In the anæmic variety the fundus is, as I have repeatedly ascertained, of a pale rose hue—a circumstance only to be explained upon the hypothesis of a diminished amount of blood in the cerebral vessels of that side. Besides this, it will almost invariably be found that if, in a doubtful case, pressure be made on the carotid artery of the side corresponding to that on which the pain in the head is felt, the pain is increased if the attack be of the anæmic form; while if it be of the congestive type, the suffering is immediately mitigated. Of course it is very essential that a correct diagnosis be made, for on that depends the kind of treatment to be administered.

Now, if it be satisfactorily determined that the patient is suffering from the anæmic variety of migraine, I at once administer a drop of the solution of nitro-glycerine of the strengsh of one-hundredth, as just described. In fifteen minutes, if relief be not obtained, I give another drop. I have very rarely had occasion to give a third dose, for amendment has gradually begun with the first drop, and a second dose almost invariably completes the cure of the paroxysm.

Attacks of migraine are generally periodical, though often in persons subject to them they may be excited by various causes, such as indiscretions in diet, emotional disturbance, or severe physical exertion. When the time comes round for a paroxysm to occur, the patient should, two or three days before it is due, begin to take the nitro-glycerine solution in doses of a drop three or four times a day, and continue the use of the remedy for a like period afterwards. When an attack is threatened from any of the causes mentioned, a like course should be pursued.

It is rarely the case that the two varieties occur in the same person, and hence when the anæmic form has been diagnosticated, it is safe to assume that all subsequent paroxysms or attempts at paroxysms will be of the same kind.

I have treated many cases of the anæmic form of migraine with nitro-glycerine, and though I cannot say that it has uniformly been successful, I know of no one agent so well calculated to give good results, and no one from which good results so generally flow. In one very severe case occurring in a lady of this city, the effect was so striking that it seemed to come from some occult influence (seemed so to her at least.) She lay in the bed in complete darkness; not a sound was allowed to come near her chamber, for light and sound greatly intensified her suffering; her skin was cold and clammy: her face pale, and the pain that racked her head was so agonizing that at times her mind wandered. One drop of the solution of nitro-glycerine was given her, and in fifteen minutes the spell was broken. Another drop, and the pain was gone; her face was flushed, and her feeble pulse was replaced by one of strength and fullness. She was well but weak. A sound sleep of three hours followed, and then she was as well as she ever was in her life.

I did not intend to cite any cases, but this one occurred in my practice while I was writing this paper, and it was so apposite that I could not refrain from adducing it, though it is not essentially different from many others.

But I do not yet definitely know that nitro-glycerine will, in every case, effect a complete cure of migraine, for the time is yet too short for such knowledge to be obtained. All to whom I have administered it continue to take it. There is every reason, however, for thinking that eventually the habit will be overcome and that the organization of some, at least, of the patients will be so modified that the disease will no longer be possible.— *Virginia Medical Monthly.*

